# Solidarity, Heterarchy, and Political Morality

**DOI:** 10.1007/s42439-020-00019-w

**Published:** 2020-06-09

**Authors:** Massimo Fichera

**Affiliations:** Faculty of Law, University of Helsinki, Helsinki, Finland

**Keywords:** Constitutional pluralism, Supremacy, Legal positivism, Transnational law, Legal authority

## Abstract

This article claims that, despite its ambivalent relationship with the heterarchical paradigm, *A Union of Peoples* is a truly innovative contribution to the complex debate on the European project, especially in the current troubled climate. Its ability to dismantle the prevailing positivist understanding of the interaction between legal orders and to stand out from the overwhelming and often repetitive literature on the philosophy of EU law should be praised. What is especially noteworthy is the idea of “corrective justice.” This notion explains very well the adoption of financial assistance measures as expression of a new form of solidarity, based on the notion of fair redress for a committed wrong, namely the structural deficiencies detectable in the design of the eurozone.

## The Definition of a (New?) Legal Entity

Elaborating a brand-new definition of the European Union (EU) has become a recurrent endeavor in contemporary legal theory. This ought not to surprise the careful and informed reader. The implications of a deep inquiry into the nature of a new legal creature such as the EU—including the question whether this is really a new creature—go beyond the object of that inquiry and concern the very nature of law. Does it still make sense to employ legal concepts and paradigms that were devised in a significantly different socio-historical landscape, largely in the nineteenth and twentieth century? Pavlos Eleftheriadis’ new book, *A Union of Peoples*, displays the adequate theoretical ambition—given the difficulty of the topic—without losing sight of the practical aspects of a fully fledged philosophy of EU law. He provides a name for such theory: *progressive internationalism*. The book relies upon two main arguments. The first argument is that, contrary to most approaches—which employ constitutional law tools and look at the EU as a federation or quasi-federation—the EU should be viewed as a model of international cooperation. In other words, the most appropriate tools to analyze the EU do not belong to constitutional law, but to (public) international law: the EU at the same time enables power sharing and recognizes the equal sovereignty of the member states.

A second, related argument that is put forward by Eleftheriadis is that law ought not to be conceptualized as a body of hierarchically ordered rules, grounded in social facts. The correct way of looking at a legal order is, in his view, constructivism or practical theory. This means that a legal order is the result of a form of moral practical reasoning, which is performed on the basis of the principle of the equal treatment of persons as free citizens and has as its object historical decisions and legal materials. Make no mistake: the target of criticism is none other than legal positivism itself, which is unable, according to Eleftheriadis, to discern between domestic and international law (as both are assumed to have the same empirical foundation) and is thus led to picture conflict between them as an inevitable occurrence, resolved by giving priority either to the EU rule of recognition over the national rules of recognition or the other way around. In reality, conflict is only in the eyes of the observer. There cannot be any competition between (public) international law and domestic constitutional law, which rely on different moral judgments, concerning inter-state cooperation in one case, the legitimacy and justice of the constitutional settlement, in the other. Hence—the author concludes—the EU is at the same time a “union of peoples” and a “community of principle.’

These two interlaced arguments will be considered more closely in the following sections. The concluding sections will instead provide a critical analysis of the author’s claims, by comparing them to alternative visions presented in analogous books published recently.

## Progressive Internationalism

As noted earlier, the author believes that the EU, far from giving birth to a radically new legal order, merely heralds a new form of *international* law. Of course, if international law is about the relationship among states, one may ask in what sense internationalism as epitomized by the EU is, as the author defines it, *progressive*. The answer is clear: what makes the European project distinctive is that, rather than purporting to build a constitution for a new state, the EU aims to ensure the mutual recognition of the moral standing of each member state. Yet, Eleftheriadis refrains from configuring the EU as a “demoicracy”—as some scholars, notably Nicolaïdis or Bellamy^1^, have done in the past—because in his view, there is no unified will behind the EU’s actions. Importantly, the form of internationalism advocated by the author does not purport to place itself mid-way between federalism and statism. It rather opposes both of them. True, the member states of the EU have come together in a spirit of reciprocity and cosmopolitanism: yet, this does not imply any form of commitment to setting up in the future either a federal union or a constitutional community. The occurrence of this event is not excluded a priori—when the conditions for a political mobilization of the people obtain—but no mandate in this sense can be currently inferred from the Treaties.

In light of this understanding, the relationship between national and EU law is regulated by three constitutional principles: conditional primacy, structural tolerance, and integrity. Conditional primacy concerns the effectiveness of EU law and ensures the validity of national law even when conflicting with EU law: in such cases—tellingly, for those who oppose the federalist view—the domestic judge merely sets aside, but does not invalidate, the conflicting national legislation. Structural tolerance ensures the validity of EU law even when it does not correspond to the interpretation of the Treaties provided by member states. Integrity, instead, consists in an obligation of consistent interpretation, which demands that EU law becomes a coherent and principled body of rules, in harmony with the domestic constitutional frameworks. Thus, ultimately the coherence of EU law is not achieved by uniformity, imposed by a single overarching master rule, but by consistency. In other words, the principle of constitutional differentiation makes it possible to coordinate the laws of the member states on the basis of similar, but not identical, constitutional principles on the division of competences. Needless to add, such principle of differentiation is a principle of international law because its moral grounding is to be found not in a doctrine of equal citizenship, but in the principle of reciprocity, as expressed by the maxim *pacta sunt servanda*. Moreover, as a result of constitutional differentiation, EU law is informed by three fundamental principles: accountability, liberty, and fairness. These principles illustrate the nature of the EU as a “union of peoples” and a “community of principle.”

First, EU law is deployed in the domestic courts as a test of accountability for every member state: this is why, as stated by the Court of Justice of the European Union (CJEU) in its case law, the Treaty creates rights, which are self-executing and which individuals are able to enforce against member states (the so-called doctrine of direct effect). Yet Eleftheriadis, contrary to most scholars in the field (and contrary to what the CJEU itself has repeatedly ruled), believes that the case law of the CJEU—starting from the landmark ruling *Van Gend en Loos*—does not indicate that a “new legal order” has emerged. The reality is that the effect of the Treaties in domestic law ultimately depends on the constitutional provisions of each member state: direct effect thus simply confirms that two distinct legal orders exist, the domestic and the international. Second, the principle of liberty removes barriers and forms of discrimination for those who move from one member state to another. EU citizenship is a central feature, but once again it should not be construed as an ordinary form of state citizenship because it aims at equal treatment on the basis of reciprocity, rather than uniform treatment. Third, the principle of fairness can be certainly associated with solidarity. However, although obligations of solidarity are particularly intense in the EU, they do not originate in distributive justice—which follows the logic of fair distribution of resources—but rather in corrective justice—which follows the logic of fair redress for unfair wrong. In other words, according to this Aristotelian mind frame, there is no point in insisting on the application of social justice to the EU, given that there are neither common political institutions setting out distributive criteria or wielding spending power, nor a demos providing legitimacy to any form of transfer of funds. In other words, fairness is not a vertical, but a horizontal principle giving rise to a duty of mutual aid: once a cooperative agreement is struck, those who bear unfair costs should be compensated by those who produced them. Corrective justice vindicates the measures enacted to address the eurozone crisis. It means that transnational (not constitutional) solidarity derives from the fact that the member states are jointly responsible for the asymmetric risks generated by the flawed architecture of the Economic and Monetary Union.

## Legal Order as the Result of Moral Practical Reasoning

The second general argument formulated by Eleftheriadis is appealing to those who are keen on investigating the nature of contemporary law. His theory of law certainly allows the reader to have a more thorough understanding of the background of his interpretation of the EU legal order. Notably, Eleftheriadis contends that a legal order ought not to be seen as hierarchically structured, but as a process of continuous interpretation and deliberation. Here, the author does not hide his inspiration mainly from the work of Ronald Dworkin, not only in the field of international law^2^, but also more generally on justice and law^3^. He occasionally also refers explicitly to the theories of Rawls, Kant, and Montesquieu^4^. As a result, Eleftheriadis blames legal positivist approaches for viewing the relationship between domestic and international law through the prism of conflict. In fact, the idea that conflict between legal orders cannot be technically conceived was at the heart of Kelsen’s theory of international law. For Kelsen, depicting international and domestic law as simultaneously valid legal orders, subject to possible conflicts, led to a logical contradiction because there can only ever exist one valid basic norm at any one time^5^. In light of this, the relationship between international law and domestic law could only be interpreted from the viewpoint of either international law or domestic law. Kelsen’s conclusion was famously monist, namely that the national legal order’s validity derives from international law, as part of one single system: this was in his eyes the only solution that allowed (and simultaneously delimited) the territorial coexistence of states, their succession in time and the exercise of their competences, i.e., the spatial, temporal, and material validity of the domestic legal orders^6^. Hart’s solution was instead that of claiming that international law is not a legal system, but a set of rules, which derives its force from practice, rather than from a union of primary and secondary rules^7^. The legal positivist account has influenced many popular approaches on the interplay between EU law and domestic law. Eleftheriadis argues that radical pluralism^8^, in particular, is indebted to legal positivism because it considers law as a technical order of rules with a rule of recognition situated at the top. According to radical pluralism, EU law is a new, hierarchically structured, legal order and, as a result, it possesses its own rule of recognition. Because the domestic and the European legal order overlap, they advance competing claims of spatial, temporal, and material validity. Hence, conflicts between rules belonging to different legal orders will inevitably concern the presuppositions of validity of the legal orders themselves. However, in the face of such conflicts, which result in overlapping monisms, the solution is to be found outside law.

The account offered by Eleftheriadis is different. As noted earlier, he belongs to a Kantian-Dworkinian line of thought, whereby the rational basis for obligations of reciprocity and solidarity is the priority of the right over the good. Human beings are equal and autonomous agents and as such they all possess dignity and equal moral standing. Public institutions emerge from the need to respect the innate right to freedom of individuals and, as a result, to recognize their rights and duties. In the same vein, Rawls believes that, given the pluralism of the good as an inescapable fact of life, our moral principles ought to be in a state of reflective equilibrium with our moral judgements. The same principles of universality and reciprocity ought to apply beyond the state borders. This is why Dworkin, too, identified the ideas of mutual respect and peaceful coexistence of sovereign states as the basis of global institutions and international law. Rather than as a hierarchical order of rules, international law should be seen as law in the interpretive, deliberative sense through the prism of political morality. What matters is not the form, but the substance of a legal system. This means that the purpose of any activity of interpretation of international rules should be that of remedying the flaws of the Westphalian system^9^. Following this approach, Eleftheriadis contends that EU law should be seen as none other than a developed form of international law, which cannot be properly analyzed either through the old versions of monism, or through radical pluralism, but rather in light of dualism. In other words, there is no hierarchy between international law and state law, because they are different orders with distinct tasks. While domestic law is the law of a jurisdiction, namely a rule-bound procedure of decision-making under general or universal laws, international law is the law that applies among nations. International law cannot be a jurisdiction, because if it were it would subsume all domestic jurisdictions. As noted earlier, this understanding is confirmed, according to the author, by most rulings of the CJEU and national courts.

## Heterarchy v. Hierarchy

The philosophical debate on the nature of the EU is rather complex and heterogeneous. For the sake of simplicity, I will distinguish between the “hierarchical” and the “heterarchical” paradigm^10^. The hierarchical paradigm considers legal orders as hierarchical structures. As the pinnacle of this structure may be located either in the domestic, or in the EU legal order, the paradigm may be respectively “State-centric” or—in various degrees—federalist. The former believes that states’ authority still carries, in one way or another, significant weight in the architecture of the EU. This view is often espoused by both traditional constitutional lawyers and international lawyers^11^. According to the federalist view, instead, the EU stands out as a coherent order, in which dual sovereignty is already operative—on the one hand, the national apparatus and the organs that represent the member states in the EU decision-making process, i.e., the council, the European Council, and the national parliaments, and on the other hand, the European Commission and the European Parliament as the organs that represent the EU^12^. In contrast, the heterarchical paradigm asserts, either as a descriptive or a normative statement, or both, the existence of multiple legal orders exercising claims towards ultimate legal authority within the same space: both constitutional pluralism and other non-hierarchical configurations of the interaction between legal orders belong to this group of theories^13^.

The book *A Union of Peoples* can thus be usefully compared to four other books that have recently been published on the same topic. They are the following: Robert Schütze’s *European Constitutional Law*^14^, Dieter Grimm’s *The Constitution of European Democracy*^15^, Tom Flynn’s *The Triangular Constitution*^16^ and my own *The Foundations of the EU As A Polity*^17^. I will begin by analyzing these books and, in a second stage, proceed to assess Eleftheriadis’ book.

Schütze and Grimm’s works are representative of the two prongs of the hierarchical paradigm, i.e., respectively the federalist and the state-centric approach.

Indeed, although Grimm recognizes the importance of the process of constitutionalization led by the creative case law of the CJEU^18^, he is not prepared to accept either the court’s excessive influence at the expense of the council, the European Parliament and national parliaments, or the un-political nature of the process of integration^19^. To Grimm’s eyes, constitutionalization amounts to depoliticization, in the sense that what becomes subject to constitutional regulation is taken away from political bargaining^20^. In particular, it is the court’s expansive interpretation of the Treaties and neoliberal economic agenda has allowed EU law to penetrate areas of competence previously covered by the member states, well beyond the goal of the internal market and often to the detriment of national fundamental rights^21^. As no constituent power may be detected at the EU level, but only at the domestic level, any interpretation of the Treaties in the sense of enlarging the powers conferred on the EU by the member states would be an amendment *contra legem*: it would threaten national sovereignty and *Kompetenz-Kompetenz* and must therefore be sanctioned by national constitutional courts (typically, the German *Bundesverfassungsgericht*)^22^. Arguing otherwise is for Grimm impossible, as the EU simply lacks the societal preconditions that allow self-legitimation and self-sufficiency. Similarly to Eleftheriadis, thus, Grimm too believes that the Treaties are products of international law, not constitutional law—and, consequently, of state sovereignty, rather popular sovereignty. Again like Eleftheriadis he, too, dismisses Habermas’ argument that a constituent power exists, in the sense that citizens operate in their dual capacity as EU citizens and national citizens^23^. The problem for Grimm is that, although the Treaties are couched in constitutional language, they do not limit themselves to enumerating the EU’s goals, powers, and procedures, but embrace a number of regulations and policy provisions, which in a domestic legal system would simply be incorporated in statutory law^24^. As a result, the commission may implement them without either the parliament or the council having a say.

Schütze’s federalist position is diametrically opposed to Grimm’s^25^. In classic Kelsenian fashion, he considers the EU Treaties as the new *Grundnorm* and asserts that there has been a shift from dual federalism—in which the federal government and the state government are sovereign coequals—to cooperative federalism—where both entities interact in a shared legal sphere. In the same vein, he considers constitutional pluralism as a misnomer because it simply describes a state of affairs which is not too dissimilar from the historical development of US federalism.

I believe that both prongs of the hierarchical paradigm miss key elements of the EU conceptual framework. On the one hand, the attempt of federalism to secure an authoritative foundation for the EU system, either by proposing a federal state or a federation of states as a *finalité*, leads to invoking a final source of legal authority—for example, through a Constitutional Treaty—which threatens the project itself by circumscribing its democratic credentials. The source of legitimacy for transnational integration seems to be identified ultimately through a top-bottom, rather than bottom-top, perspective. Local revindications, idiosyncrasies, and impermeability are too often downplayed or ignored. On the other hand, as regards state-centric approaches, the overall feeling is that, however conceived, they fail to capture—as will be discussed below—the constitutional relevance of the self-justifying moves made by the CJEU, the national courts and other European institutions, such as the European Commission.

More convincingly than the hierarchical paradigm, the heterarchical paradigm either downplays the importance of seeking a final authority or avoids invoking such authority altogether. No hierarchical supremacy of one legal system over the other can be established a priori: neither monism nor dualism quite grasp the complexity of the interactions between multiple, overlapping, and interlocking normative systems in contemporary world^26^. A recent account belonging to this third group of theories is Flynn’s *The Triangular Constitution*. Constitutional pluralism—and its critics—comes in a considerable variety of forms. Yet, Flynn offers an interesting explanatory framework, whereby the legal orders of the EU, the European Conventions of Human Rights and Ireland are part of a composite, polyarchic order.

However, the heterarchical paradigm sometimes—especially when it elaborates principles of best fit or coherence—ends up locating the ultimate source of authority at the EU level and does not cut loose neatly and unambiguously from its moorings in the domestic configuration of a constitutional order^27^. Thus, I believe that the most promising versions of the heterarchical paradigm are those which adopt an epistemic perspective. Accordingly, they contend that, given the irreconcilability of the claims to final authority put forward from within each legal system, the question of the final authority should be left open—while at the same time devising a set of meta-constitutional rules that can be employed to either bracket conflict or find a—however temporary—solution^28^. Flynn’s model firmly situates his work in this context^29^. Yet, it also claims, somewhat differently that the interface norms regulating the relationship between legal orders do not exist a priori, but only emerge on a case-by-case basis and evolve over time—hence the evolutionary nature of the tripartite deliberative polyarchy resulting from the interplay and deep intertwinement of non-state constitutional orders^30^.

Clearly, Elefhteriadis sets himself apart from mainstream theories of EU law, as exemplified by the scholars mentioned above. Although he shares Grimm’s skepticism towards any sort of transnational constituent process, he is not equally prepared to speak the language of constitutionalism (especially when it comes to *Kompetenz-Kompetenz* issues). His vision of law as moral judgment is directed against precisely the kind of systemic thinking that characterizes Schütze’s work (and others). His relationship with the heterarchical paradigm is more ambivalent, as he himself claims to follow a heterarchical approach.

In light of the above, the inevitable question is: does *A Union of Peoples* fit within the heterarchical or the hierarchical paradigm? First of all, Eleftheriadis does not target all versions of this paradigm, but mostly radical pluralism and his main proponent, MacCormick, who is criticized for still relying on the Kelsenian idea of constitutional authority as a hierarchy of rules. Here, the author does not consider that MacCormick’s theory is not entirely “pure,” but rather ‘institutional’. Although MacCormick aims to keep separate the “is” from the “ought,” he emphasizes inter alia the function of the law as a means of social integration, and the principled nature of democratic legal orders^31^. By way of contrast, *A Union of Peoples* constructs its argument and develops its epistemic claims purely in the domain of law. In this regard, it is telling that Eleftheriadis dismisses any sociological account of law, i.e., the idea that law is what people identify as such through social practices.

Second, surely it cannot be ignored that Eleftheriadis rehearses a theory—dualism—which was elaborated at the end of the nineteenth century by positivist scholars who considered international law’s validity as ultimately premised upon state will. This is noteworthy. It helps providing an answer to the hierarchy/heterarchy question. Although he does not consider himself positivist, his theory presents many affinities with the hierarchical paradigm, precisely because the choice whether or not to incorporate “foreign” law is left entirely to the domestic sphere. States are ultimately sovereign in their own territory, yet cooperate with each other for moral reasons.

I share Eleftheriadis’ concern for moral judgment. In my own work, I argue that the European project is founded upon the meta-constitutional rationale of security as self-preservation, which informs simultaneously the process of transformation of the EU polity, the practices and institutional arrangements that characterize it, and the scholarly work on EU integration. Security thus corresponds to a sort of Dworkinian political morality and reflects the Kantian ideal of reconciling freedom with coercion^32^. Because founding a polity means also attempting to secure its long-time survival, security, whose essential features are thus existential, lies at the heart of Europe’s constitutional identity and reveals itself through two self-justifying discourses of power^33^: security and rights. Although these discourses are constitutive of the European project at a deep, foundational level, they are characterized by ambiguities and contradictions and have appeared as neutral—thus concealing legal-political conflict^34^. In fact conflict, contradictions and the interplay between security and crisis are essential features of European *thin* form of constitutionalism, especially as it moves towards more advanced stages^35^. In light of this, I claim that the main question currently should be not so much what the nature of the EU is, but *what for* or *why* European integration is taking place^36^. The main dilemma facing the European project is that its unyielding perseverance in pursuing a specific goal at all costs may impair the very ideal which inspires that project. Hence, I advocate a move from self-referential to heterarchical security, i.e., from a form of cooperation based on one-size-fits-all models to a multi-layered framework, in which concern for the local, national and sub-national level, and for social and economic integration is combined with an effort to protect and promote the core values of the European project over an extended period of time^37^.

It follows that I also agree with Eleftheriadis’ argument that the EU’s roots are to be found in international law and a federalist vision of Europe does not correspond to a realistic scenario. However, contrary to Eleftheriadis, I argue that the EU has evolved into a novel form of *transnational* law, with “thin” constitutional implications. The transnational nature of the EU is also associated with the displacement of the traditional public/private divide and the related configuration of the public sphere also through horizontal relationships between private (and, in general, non-state) actors. There are many reasons for this conceptualization. First of all, as Flynn notes in his work, legal claims of authority overlap because they have become functional, and not only territorial. Second, the EU legal order is characterized by complex networks, agencies, soft legislation, which, unlike the classical international organization, are very effective in penetrating into the domestic sphere. Third, viewed from a dynamic, not static, perspective, European integration is *constitutional* integration, in so far as it relies upon a form of *discursive* constituent power, whereby it constructs a dual conception of “The People” —as “mobile people,” i.e., EU free movers, and as “peoples” in the plural—precisely through security and rights discourses^38^. Discourses develop at all societal layers: yet in particular those unrolling in the case law of the CJEU and national courts—especially when they employ constitutional vocabulary—should not be dismissed too easily, as they reflect a level of intersubjective awareness and perception of the implications of European integration, which is deeply embedded in large sectors of the population. Curiously, the author is happy to employ case law that proves his dualist approach, but looks at those cases that point in another direction as “exceptions.” In this sense, the elaboration of the idea of citizenship and the self-constitution of the EU as an “area of freedom, security and justice”—not as a specific policy branch, but as an overarching aspiration of the European project—surely have important repercussions on the development of the EU as a legal and political system. No doubt, then, the role of courts and the symbolism attached to the image of “The People”—as illustrative aspects, respectively, of the rule of law and of the democratic component of a polity—are necessary ingredients of a dispassionate account of the degree of development of that very polity.

Of course, on the one hand, court-centric approaches should be viewed with suspicion. From this point of view, Eleftheriadis’ and Grimm’s skepticism towards judicial supremacy is understandable: yet their argument does not take into account—as Flynn does—the potential of deliberative polyarchy as a process of constant, cyclical inter-institutional dialog within and across multiple legal orders—a dialog in which courts are significant, but by no means the only players^39^. On the other hand, Eleftheriadis’ emphasis on a union of *peoples*—in the plural—is key to an appropriate framing of the contours and object of the debate in terms of *both* pluralism *and* plurality. As already discussed, Eleftheriadis claims that, because law is a set of principles protecting the rule of law for moral purposes, the solution to conflicts can only be legal. Analogously, in this case, to many versions of the heterarchical paradigm, *A Union of Peoples* downplays the impact that legal-political conflict and the interplay between security and crisis have for the European project. The existential character of security has been key to crucial (judicial and non-judicial) polity-building steps, often *in the name of the EU peoples*. According to Eleftheriadis, the CJEU’s doctrine of the “new legal order” entails a legal revolution and thus cannot be legally valid. This reasoning is based on the binary code legal-illegal: change is only conceived in one sense or the other. However, change can also occur as an a-legal phenomenon, and it is curious that Eleftheriadis, while rejecting Kelsen, still relies on his configuration of legal validity. Ultimately, the book’s criticism is very effective when applied to either federalist or state-centric approaches, but less so as regards radical pluralism.

Despite its ambivalent relationship with the heterarchical paradigm, *A Union of Peoples* is a truly innovative contribution to the complex debate on the European project, especially in the current troubled climate. Its ability to dismantle the prevailing positivist understanding of the interaction between legal orders and to stand out from the overwhelming and often repetitive literature on the philosophy of EU law should be praised. What is especially noteworthy is the idea of “corrective justice.” This notion explains very well the adoption of financial assistance measures as expression of a new form of solidarity, based on fair redress for a committed wrong, namely the structural deficiencies detectable in the design of the eurozone—although one may wonder whether a different form of solidarity, of a more distributive nature, would be required when no form of culpability can be detected, as is the case with the difficult negotiations for the adoption of appropriate health and fiscal measures in order to alleviate the consequences of the economic downturn caused by the spread of the coronavirus. In fact, both the eurozone and the coronavirus crisis have showed how the high degree of socio-economic interdependence achieved by the EU makes it inevitable to ensure some form of fiscal burden-sharing and mutualization of debts^40^ precisely in order to address critical situations that threaten the existence of the European project. This could mark a constitutional moment for the European project and a step further towards a full heterarchical paradigm. National courts, acting as EU courts, ought to be given a more prominent role, especially when it comes to the interpretation of EU law according to their own legal-cultural parameters. The political, inherently conflictual nature of EU constitutional claims—which has been concealed for too long beneath a veil of neutrality—becomes more evident as European integration reaches more advanced stages. In this context, the role of the EU judiciary in the self-preservation and self-perpetuation of the EU legal order is much more relevant than might seem at first sight. Yet, because of their political nature, the solution to conflicts between legal orders—as the one triggered by the ruling of the German Bundesverfassungsgericht in 2 BvR 859/15 a.o. of May 5, 2020 on the European Central Bank’s program of quantitative easing—requires a certain degree of cooperation between judicial and non-judicial organs.

## Footnotes

1 Nicolaïdis 2012; Bellamy 2013.

2 Especially Dworkin 2013.

3 Typically, Dworkin 2003, 2006, 2011.

4 Kant 1999a; Kant 1999b; Kant 1999c. See also Rawls 1999a, b; de Montesquieu 1989.

5 Kelsen 2005, 328–329. This is an epistemic claim: the principle of non-contradiction must be the presupposition of legal cognition.

6 Kelsen 2005, 336. For a thorough treatment of Kelsen’s view, see e.g., Vinx 2007. In fact, Kelsen in other works also held the perspective of *national* monism.

7 Hart 1993, 232–236.

8 The most famous scholar, indeed the founder, of this approach is Neil MacCormick. See MacCormick 1993; MacCormick 1995; MacCormick 1999.

9 Dworkin 2013, 22.

10 This distinction only partly recalls Walker’s distinction between “particularism” (or “philosophical monism,” which includes both monism and dualism because it locates the ultimate choice of how to treat foreign law in the state legal system) and “holism”, which rejects systemic thinking in law and opts for a holistic framework of political morality that downgrades or dissolves the relevance of political disputes. Walker however does not consider federalism as “particularist.” Moreover, “holism” does not necessarily correspond to my configuration of “heterarchy.” See Walker 2016.

11 See, among many, Grimm 2017; de Witte 2017.

12 Typically, Schütze 2016.

13 As regards constitutional pluralism, see e.g., Kumm 1999; Walker 2002. Another example of the heterarchical paradigm is Gerstenberg 2018.

14 Schütze 2016.

15 Grimm 2017.

16 Flynn 2019.

17 Fichera 2018.

18 Grimm 2017 5.

19 Grimm 2017 10.

20 Grimm 2017, 128–129.

21 Grimm 2017, 29–30. Grimm’s skepticism towards the CJEU is somehow akin to the dismissal of counter-majoritarianism that is typical of political constitutionalism. See e.g., Bellamy 2019.

22 Grimm 2017, 53–55.

23 See e.g., Habermas 2012.

24 Grimm 2017, 73.

25 Schütze 2016, 43–76.

26 For an early version of dualism, see von Triepel 1899.

27 I discuss this in Fichera 2018, 2.

28 Among many, Walker 2000.

29 Flynn 2019, 25.

30 Flynn 2019, 149–150.

31 MacCormick 2007. MacCormick later moved to a milder position of “pluralism under international law,” which he himself described as Kelsenian: MacCormick 1999, 121.

32 Fichera 2018, 166. However, the configuration of the security meta-constitutional rationale owes much also to the theories of *raison d’état* (e.g., Machiavelli). All relevant principles of EU law, including primacy, effectiveness, and uniformity, as well as the notion of common constitutional traditions, are expressions of security.

33 ‘Discourses’ include formal and informal social practices and interactions between economic and social actors (e.g., courts, parliaments, media, academic work, as well as social movements, and trade unions), i.e., not merely “groupings of utterances or statements,” but “whatever signifies or has meaning” and produces effects within a social and institutional context.

34 The reference is in particular to the development of the internal market as a supposedly neutral endeavor.

35 I also argue that, for the first time in the history of European integration, all dimensions of security—spatial, temporal, popular, ontological, epistemic, and reflexive—have been affected by the multiple crises in the last years.

36 This is in line with the idea of “law as a purposeful enterprise”: see e.g., Fuller 1964, 145. See also Fichera 2018, 23.

37 Fichera 2018, 181.

38 Fichera 2018, 39–63.

39 See e.g., Gerstenberg and Sabel 2002.

40 Fichera 2018, 165.
